# Inflammatory Cytokine Pattern Is Sex-Dependent in Mouse Cutaneous Melanoma Experimental Model

**DOI:** 10.1155/2017/9212134

**Published:** 2017-11-26

**Authors:** Mihaela Surcel, Carolina Constantin, Constantin Caruntu, Sabina Zurac, Monica Neagu

**Affiliations:** ^1^Immunology Department, “Victor Babes” National Institute of Pathology, 99-101 Spl. Independentei, 050096 Bucharest, Romania; ^2^Faculty of Biology, University of Bucharest, 91-95 Spl. Independentei, 76201 Bucharest, Romania; ^3^Colentina University Hospital, 19-21 Stefan cel Mare Blv., 020125 Bucharest, Romania; ^4^“Carol Davila” University of Pharmacy and Medicine, 37 Dionisie Lupu Street, 020021 Bucharest, Romania

## Abstract

We present the evaluation of inflammatory cytokines in mouse cutaneous melanoma experimental model, as markers of disease evolution. Moreover, to test our experimental model, we have used low doses of dacarbazine (DTIC). C57 BL/6J mouse of both sexes were subjected to experimental cutaneous melanoma and treated with low doses of DTIC. Clinical parameters and serum cytokines were followed during tumor evolution and during DTIC therapy. Cytokine/chemokine pattern was assessed using xMAP technology and the following molecules were quantified: interleukins (IL)-1-beta, IL-6, IL-10, IL-12 (p70), interferon (IFN)-gamma, granulocyte macrophage colony-stimulating factor (GM-CSF), tumor necrosis factor (TNF)-alpha, macrophage inflammatory protein (MIP)-1alpha, monocyte chemoattractant protein (MCP-1), and keratinocyte-derived chemokine (KC). Significant differences were found between normal females and males mice, female mice having a statistically higher serum concentration of IL-1-beta compared to male mice, while males have a significantly higher concentration of MIP-1-alpha. During melanoma evolution in the female group, IL-1-beta, MIP-1-alpha, and KC circulatory levels were found 10-fold increased, while other cytokines doubled their values. In the male mice group, only circulatory KC increased 4 times, while IL-1-beta and TNF-alpha doubled their circulatory values. Various serum cytokines correlated with the disease evolution in cutaneous melanoma mouse model.

## 1. Introduction

Cutaneous melanoma, one of the most aggressive human cancers, is a subject of intense research and constant discoveries [1]. Melanoma, as in many other types of solid tumors, expresses cells and molecular features of inflammation and an array of inflammatory cytokines that follow this disease. There is a subtle battle between the pro- and anti-inflammatory actors [2]. Thus, during an acute inflammatory response, innate cells produce mediators that attract and trigger T-helper (Th)1-polarized T lymphocytes, to secrete cytokines with antitumor activity (e.g., interleukin (IL)-2 and interferon (IFN)-gamma). T cells in combination with antitumor-directed B cell-derived factors (e.g., immunoglobulins) activate tumor inhibitory responses by recruited innate immune cells and cytotoxic T lymphocytes (CTLs); all this cell's *army* can induce a tumor rejection. In contrast, when there is a chronic activation of immune response without resolution of the damaged tissue, accumulation of regulatory T (Treg) cells, Th2 cells, and activated B cells is induced; all these cells secrete protumorigenesis factors (e.g., IL-4, IL-6, IL-10, IL-13, and transforming growth factor (TGF)-beta) that enhances protumorigenesis responses in innate immune cells and inactivate CTL cytotoxicity, thus favoring tumor promotion [3]. Consequently, the mediators and cellular effectors of inflammation are, on one hand, common to tumor microenvironment and reside in the tumoral site on the other. Furthermore, inflammatory conditions can preclude a malignant transformation and/or an oncogene alteration sustains the inflammatory microenvironment favorable for tumor development [4, 5].

We will tackle herein the inflammatory markers following the cutaneous melanoma experimental evolution to discover the ones that can pinpoint the disease evolution.

Dacarbazine (DTIC), the first FDA-approved cytostatic for metastatic melanoma [6], is an imidazole carboxamide derivative with several proposed mechanisms of action [7], a cytostatic that is still in use in human therapy approaches. One of the first reported studies on melanoma mouse models has shown that DTIC proved in mouse the highest sensitivity [8, 9].

Taking into account all the mentioned issues, we have studied in a standard melanoma mouse model the soluble cytokine/chemokine pattern during melanoma progression and during low doses of DTIC therapy. Survival rate, tumor volume, and soluble cytokine/chemokine monitorization were followed up. Concomitant detection using multiplexing techniques enabled us to evaluate cytokines/chemokines that sustain the inflammatory processes associated to tumor development.

## 2. Material and Methods

### 2.1. Murine Experimental Model

In order to monitor the inflammatory process, we have developed the standard animal model for developing cutaneous melanoma [10] using C57BL/6 J mice subcutaneously inoculated with B16 melanoma cell line. Female and male C57BL/6 J mice purchased from Jackson Laboratory (Bar Harbor, ME) were maintained in standard conditions in “Victor Babes” National Institute of Pathology Animal Husbandry. The experiments were approved by the Institute's Ethics Committee, and all the approaches were in accordance with the recognized principles of laboratory animal care in the framework of *EU Directive 2010/63/EU for animal experiments* [11].

Each presented mice group consisted of 20 males and 20 females, 6 weeks of age, with a mean weight of 23 ± 2 g. Groups supposed to develop skin melanoma were subcutaneously inoculated with 1 × 10^5^ B16F10 (ECACC 92101204) melanoma cell lines/mouse. Groups that were intended for DTIC treatment in the 7th day tumor cell postinoculation were treated intramuscularly with low doses of DTIC (5 mg/kg/mouse) for 5 days at 24 h intervals. Mice were retroorbitally bled at day 0 and day 7 from B16 inoculation and DTIC posttreatment. Controls mice were bled at the same intervals. Blood was subjected to serum harvesting and afterwards stored at −70°C until testing.

The following groups were tested:

- Control (tumor-free animals)
- Control DTIC (tumor-free animals subjected to low doses of DTIC)
- Mice-bearing B16F10 melanoma tumor (untreated)
- Mice-bearing B16F10 melanoma tumor subjected to DTIC

### 2.2. Clinical Parameters

Mice were followed for tumor volume that was measured according to Egorov [12] formula: *V* = *π*/6 × (length) × (width) × (height) and expressed in mm^3^. Results were presented as mean ± SD mm^3^. The sampled tissues were subjected to histological analyses. Postmortem necropsies were performed to identify the presence and the extent of metastasis.

### 2.3. Serum Cytokine Testing Using xMAP Technology

Taking into account the low volume serum samples and the necessity to investigate a large panel of inflammatory immune-related molecules, we have used xMAP array technology. Mouse Cytokine/Chemokine Lincoplex Kit (Cat. number MCYTO-70 K) and Luminex 200™ equipment were used for quantifying the following immune-related factors: interleukins (IL)-1-beta, IL-6, IL-10, IL-12 (p70), interferon (IFN)-gamma, macrophage colony-stimulating factor (GM-CSF), tumor necrosis factor (TNF)-alpha, monocyte chemoattractant protein (MCP-1), macrophage inflammatory protein (MIP-1), and keratinocyte-derived chemokine, also known as mouse CXCL1 (KC). We have investigated both pro- and anti-inflammatory cytokines that are secreted by a large array of immune and nonimmune cells as presented in Table 1. The entire procedure was performed according to the producer's recommendations. The calibration curves were obtained using the provided standards, and the method accuracy was analyzed using the registered high and low controls provided by the kit. Quality parameters lay within the recommended ranges. Data were collected and analyzed by Luminex xPONENT software and a five-parameter regression formula was used to calculate sample concentration from the standard curves. Results are presented as mean ± SD of pg/mL serum concentrations. For statistical analysis, the Student's *t*-test was used and *p* value under 0.05 was considered statistically significant.

## 3. Results

### 3.1. Clinical Parameters

**Figure 1:**
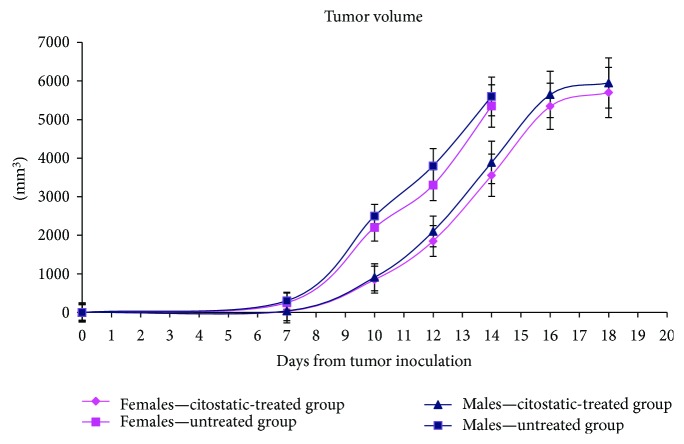
Tumor volume dynamics in an experimental melanoma model in comparison to the DTIC-treated group in female and male mice groups (mean ± SD).

**Figure 2:**
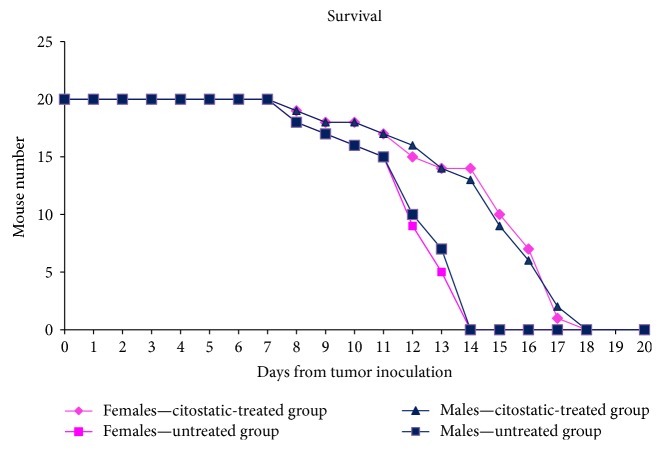
Survival dynamics of mouse melanoma model in comparison to the DTIC-treated group in female and male mice groups.

The investigated groups, for both sexes, had a similar evolution of the tumor volume. At day 7, the tumor volume of low concentration of DTIC group was lower than the untreated group (Figure 1). Therefore, as expected, the untreated group had the highest increase in tumor volume and, at day 14, from the untreated groups, all mice have died (Figure 2). The postmortem pathology has revealed massive melanoma metastases in the brain, lung, pleural membrane, liver, kidney, adrenal glands, lymph nodes, and muscles. The tumor volume of animals treated with cytostatic increased at the same rate and at day 18, all the animals from the group have died. The DTIC-treated group had an increased survival rate with a mean of 30%. The increase in the survival rate was based on lower metastatic accumulation. No significant differences were registered in terms of tumor volume or survival rates between males and females and no significant difference in distant metastatic sites.

### 3.2. Serum Cytokines in Tumor-Free Mice

**Figure 3:**
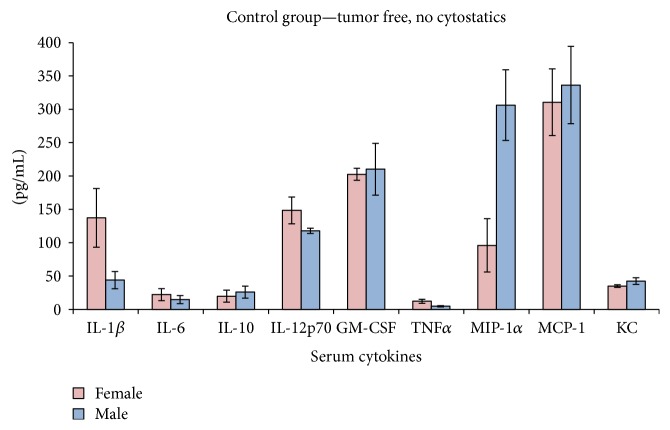
Serum cytokines/chemokines in control groups (mean ± SD).

**Figure 4:**
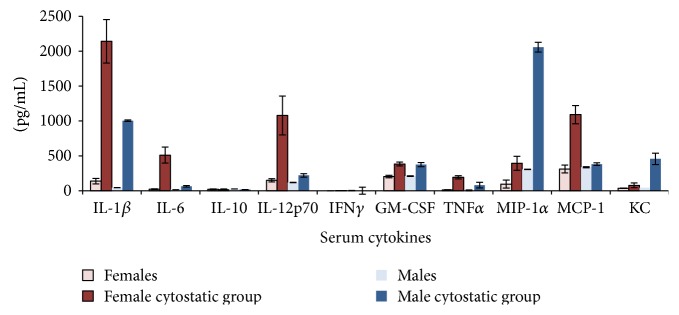
Cytokine/chemokine serum pattern in normal mice (female and male mice groups) subjected to low doses of DTIC (mean ± SD).

In control groups (tumor-free, no therapy), the pattern of circulatory cytokines/chemokines is totally different when investigating females versus males. This assertion should be taken into account when further comparing the effect of tumor development in both sexes (Figure 3). Female mice have a statistically higher serum concentration of IL-1-beta compared to male mice, while males have a significantly higher concentration of MIP-1-alpha (*p* < 0.0005). A difference in the IL-12p70 and TNF-alpha serum concentration was detected as well (*p* = 0.01). All other cytokines did not have significant differences between the two sexes. In order to investigate the proinflammatory effect of low doses of DTIC in normal mice (tumor-free), we have tested also these mice groups. Surprisingly, we have obtained in both males and females a clear statistically significant increase of the circulatory cytokines/chemokines upon DTIC treatment (Figure 4). Overall, we have obtained significantly high serum concentrations of IL-1-beta, IL-6, IL-12, GM-CSF, TNF-alpha, MIP, MCP, and KC. Although the cytostatic was used in low concentrations, it actually induced in both sexes a cytokine/chemokine storm.

### 3.3. Serum Cytokines/Chemokines Indicate Cutaneous Melanoma Development

**Figure 5:**
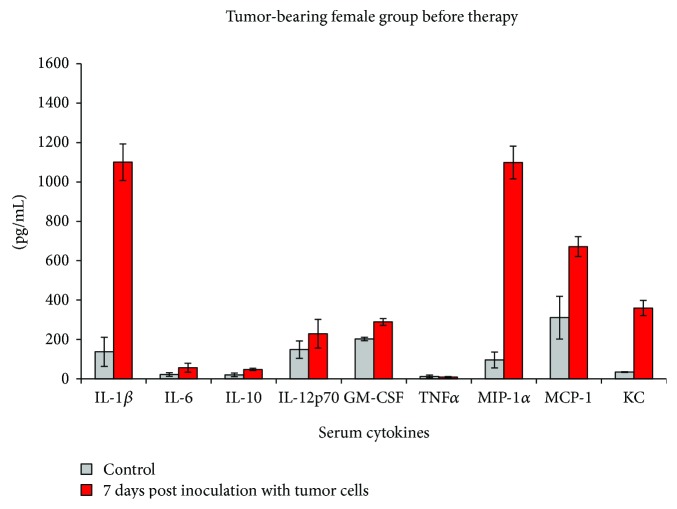
Cytokine/chemokine serum pattern in the female group after 7 days post inoculation with B16 melanoma cell line (mean ± SD).

**Figure 6:**
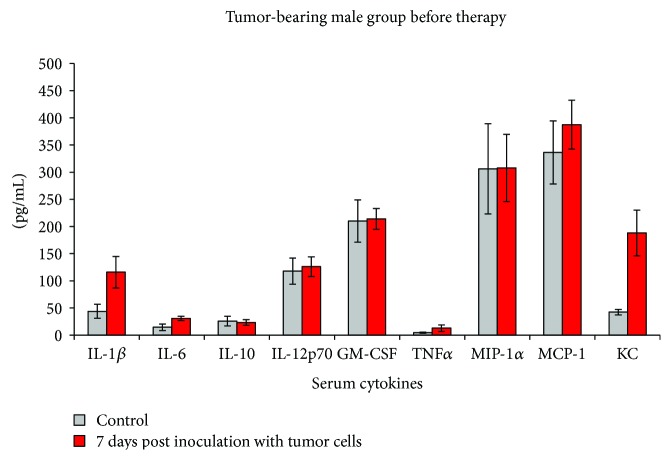
Cytokine/chemokine serum pattern in the male group after 7 days post inoculation with B16 melanoma cell line (mean ± SD).

After 7 days postinoculation of B16F10 melanoma cell line, when a detectable tumor has appeared, both male and female mice displayed higher serum concentrations of some of the tested proinflammatory cytokines (Figures 5 and 6).

In the female group, IL-1-beta, MIP-1-alpha, and KC circulatory levels had a 10-fold increase after 7 days postinoculation, while IL-6, IL-10, and MCP-1 statistically doubled their value. GM-CSF was registered with a statistically significant increase of 42%, while the increase of IL-12 was not statistically significant compared to controls as was the decrease with 30% of TNF-alpha (Figure 5).

The inflammatory pattern of male mice at 7 days postinoculation was completely different (Figure 6). We did not register for any of the tested cytokines/chemokines the same increase as in the female group. Hence, circulatory KC increased 4 times, while IL-1-beta and TNF-alpha statistically doubled their circulatory values. The increases of circulatory IL-6, as the decrease of IL-10, were not statistically different. IL-12, GM-CSF, MCP-1, and MIP-1 were practically not affected by tumor evolution in the male mice group.

As the normal female group already registered higher serum concentrations of IL-1-beta compared to the male serum, we have seen an important increase of this cytokine posttumor inoculation. After 1 week postinoculation, MIP-1alpha, MCP-1, and KC were additionally significantly increased in females. Less sensitive to B16 tumor inoculation, the male group had less cytokines/chemokines types increased; KC and IL-1-beta were the main factors found increased compared to the controls. It seems that in tumor-bearing animals, regardless of their sex, soluble KC is a parameter that is steadily increasing in the serum. One probable explanation is that melanoma cells can intensively secrete KC [13]; therefore, an ongoing tumoral process can be associated with an increase in serum KC.

### 3.4. Serum Cytokines/Chemokines during Cytostatics Treatment

**Figure 7:**
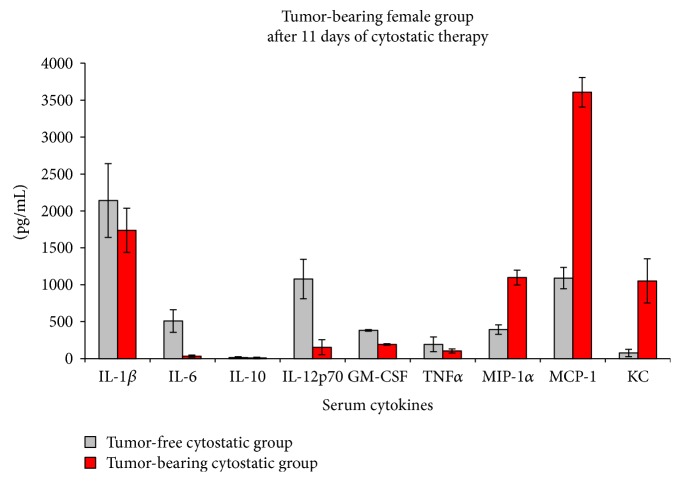
Cytokine/chemokine serum pattern in the female group subjected to low doses of DTIC (mean ± SD).

**Figure 8:**
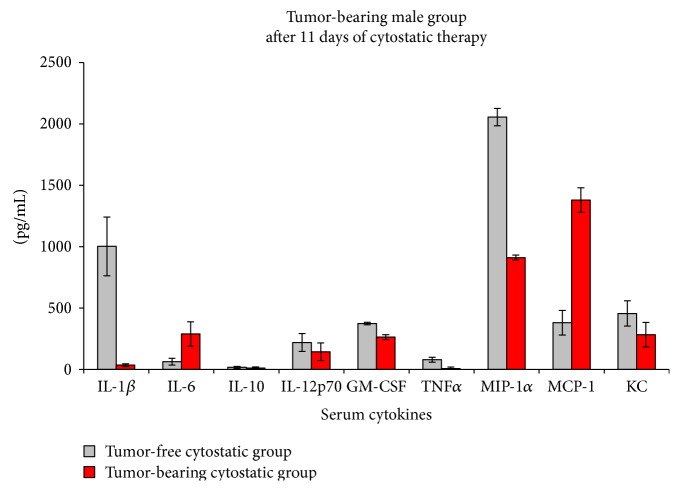
Cytokine/chemokine serum pattern in the male group subjected to low doses of DTIC (mean ± SD).

In order to test the sensitivity of the inflammatory cytokine pattern in relation to therapy, we have treated mice with low doses of DTIC. As previously stated, the cytokine/chemokine pattern is different in the female versus male mice groups (Figures 7 and 8). In the female group, there are statistically significant decreases registered in IL-6, IL-12, and GM-CSF levels and significantly increased values for MIP-1, MCP-1, and KC, these last 3 factors being 2.7, 3.3, and 13.5 times increased after DTIC treatment (Figure 7).

In the male group, the cytokine pattern is different upon low doses of DTIC treatment. Therefore, if in the female group, there is a nonsignificant decrease of IL-1-beta, in males, the decrease is important and statistically significant, registering an almost 1000-fold reduction. Along with IL-1-beta decrease, MIP-1, TNF-alpha, and GM-CSF are statistically decreased. IL-6 and MCP-1 were found significantly increased, 4.5 and 3.6 times, respectively. IL-10 and KC although have a tendency to decrease, they were found not statistically significantly changed upon DTIC therapy (Figure 8).

## 4. Discussion

The tested cytokines have different functions and intercede at different stages in tumor development. IL-1-beta is intimately correlated to inflammasomes that are responsible for the inflammatory milieu generated through cytokine production and secretion. Inflammasome assembly process induces in the end, the generation of the active form of IL-1-beta which stimulates the inflammatory response in neoplasias, such as melanoma [14]. In our experimental approach, this serum cytokine is elevated during melanoma evolution and in both animal groups, it is reduced upon cytostatic therapy. Our previously published data on human IL-1-beta detected in serum [15] have shown that starting from early stages (stage I) this cytokine has a twofold increase compared to the control. Accordingly, data published by Okamoto et al. [16] have shown that at the tumoral level the expression of IL-1-beta increases in primary tumor versus normal/benign tissue and increases once more in the metastatic tumor. Therefore, within the scope of our study, namely, in terms of monitoring disease, at least in male mice, IL-1-beta is a circulatory indicator for therapeutic efficacy.

Circulatory IL-6 was reported in both human [15, 17] and mice [18], showing serum IL-6 elevation correlated with poor prognosis in melanoma and progression towards metastasis. Our previous studies in melanoma patients have shown an IL-6 increase as a marker of immune system upregulation sustained by the keratinocytes that secrete this cytokine in order to enhance the T cells' antitumoral activity [15]. Cellular studies published last year have shown that cytokine tumor microenvironment can modulate tumor-initiating cells in melanoma. While IL-6 can induce differentiation, IL-10 supports self-renewal and other cytokines, like CCL-2 and IL-8, did not influence this activity [19]. In our mouse model, IL-6 was found different in the male group versus female group upon melanoma cytostatic therapy, while drastically reduced in females, it was found increased in males. Although in humans there are extremely sparse studies regarding male versus female IL-6 circulatory concentration and none of them are related to melanoma studies, there are publications that show lower serum values in women compared to men [20]. Besides many other explanations, one possible explanation of IL-6 sex difference that we are reporting in this paper relies on the recently reported metabolic differences in C57BL/6 J strain [21]. Hence, females have a diminished insulin sensitivity that can account for the low proinflammatory level of circulatory IL-6 [21]. In humans, we cannot exclude a similar mechanism as it was also reported that IL-6 has a modulatory activity upon insulin metabolic pathways [22, 23].

As shown in human samples [15], IL-8 could be an indicator only for late stages, and as we intended to monitor the melanoma evolution, we did not evaluate this cytokine in a mouse model.

Earlier studies have shown that the IL-10 family [24] selectively induced apoptosis in melanoma, promoting clear antitumor activity [25]. Other studies have shown that IL-10 induces its specific receptor on CD8^+^ T cells that leads to activation and antigen-specific expansion, depicting IL-10 as an emerging therapeutic molecule [26]. In humans, we found circulatory IL-10 strongly elevated in late stages of melanoma positively correlating with the circulatory elevated T lymphocyte CD4^+^ and CD25^+^ with FOXP3 expression [15]. Although IL-10 has a low serum concentration in the presented mouse model, it was found increased only in female tumor-bearing animals, but not in the male group. After therapy, although we register an alteration of the serum level, it was not statistically significant. IL-10, an anti-inflammatory cytokine can be produced by an array of immune cells but mainly by monocytes (see Table 1). Recent studies have shown that estrogen protection induces a regulatory feedback loop between M2 macrophages and regulatory B cell [27] loop that can account for the higher circulatory levels depicted by us in the female groups.

IL-12 was shown earlier to prime naïve CD8^+^ T cells developing an increased anti-tumor activity [28] and acting synergistically with TGF-beta [29]. Moreover, it was shown that the combined effect of IL-12 and TGF-beta significantly induced NK cell activity in *in vitro* systems upon cells isolated from melanoma patients [30]. Thus, in humans, IL-12 accounts for an active immune response [15]. In the present study, as in other cytokines, IL-12 statistically decreased after cytostatic therapy in the female mice group while being unaltered in the male group. This sex difference pattern can be also accounted on the IL-12 secretion related to hormone activity [31].

Melanoma tumor cell exosomes were reported to induce *in vitro* GM-CSF expression by endothelial cells favoring angiogenesis in lymph nodes [32]. When we tested this cytokine, we observed that in the female melanoma group, this growth factor is statistically increased and then, upon therapy, it decreases. Recent data show that dendritic cell differentiation is GM-CSF-dependent and it needs estrogen receptor activation. Estrogen receptor activation is furthermore needed for naive CD4^+^ T lymphocyte activation that secrete GM-CSF (see Table 1) [33].

In our system, although in both groups circulatory TNF values were found low, we have registered a posttherapy decrease of this cytokine in both groups of sexes. Recent data have shown that upon melanoma cell lines, TNF and IL-1 showed no significant overall effect on expression of molecules involved in metastatic processes. Nevertheless, arrays of cytokines/chemokines and various regulatory molecules (e.g., matrix metalloproteinases) can modulate this process [34]. Our previous human reported TNF values [15] were as well low and, as prior signaled by us, the low registered circulatory levels can account for the multiplexing assays that use specific antibodies for different circulatory molecules. Nonetheless, as serum cytokines have a huge domain concentration span (from very high to very low); thus, the lower concentrations can be hindered by the signals developed by the ones with higher and more robust concentrations. We do not rule out that, if classical ELISA quantification for individual cytokines would have been used, sex difference would have been signaled as well for circulatory TNF.

When testing in humans, the circulatory MIP-1 alpha has contradictory reports. While MIP-1 alpha was found significantly increased in melanoma patients in comparison to healthy subjects and associated with longer recurrence-free survival rates [35], others show low values of circulatory MIP-1 alpha in melanoma patients [36]. In our model, we have registered a very clear decrease of serum MIP after therapy in female mice correlated with a good evolution, while an increase in the male group after therapy. In this case, we found again a clear difference between the male and female groups in terms of cytokine pattern. As an overall comment, sex hormones play a key role in an individual's immune response, thus the immune processes can be sexually dimorphic [37]. As MIP is secreted by macrophages and was reported as tumorigenesis promoter via androgen-receptor [38], we cannot rule out in our model that this difference resides on the existence of androgen receptors in males that regulate MIP serum levels depicted by us. For example, in humans, prostate cancer can be associated with cutaneous melanoma. This assertion is sustained by the fact that the MAGE family (melanoma antigen genes-A) are expressed both in melanoma and in prostate cancer. In 2017, it was demonstrated that MAGE-A proteins can potentiate specific oncogenic processes being involved in several unrelated cancers due to the complex MAGE gene network [39, 40].

It was reported that MCP-1 favors tumor angiogenesis and early tumor growth by inducing TNF-alpha, IL-1alpha, and VEGF secreted mainly by tumor-associated macrophages [41]. Recent studies have shown that in melanoma-bearing mice, myeloid-derived suppressor cells favor tumor growth associated to elevated MCP-1 and IL-10 [42, 43]. In our model, we found that tumor-bearing female mice had an increased MIP serum level correlated with an increased MCP. The same pattern is obtained during therapy in the female group. Interestingly, in the male group, we have completely different pattern after therapy, the highest increase in MCP induced after the therapy was found in the male-treated mice group. In our model, the inflammatory process that is triggered by melanoma development (inflammation per se) can be depicted by an elevation in proinflammatory cytokines, like MCP, the inflammatory status being strongly correlated with sex steroids [44].

An interesting molecule, KC, known to influence melanoma cells [45, 46] is at the base of intercellular interactions in melanoma cell-keratinocyte tandem. Early reports showed that this molecule is also secreted by melanoma tumor cells [47]. Our finding for KC level is more so interesting when we add that, in tumor-bearing animals, this chemokine was found significantly increased in both sexes and after DTIC therapy, the level of KC considerably decreased, especially in the female group. Our data correlate with a previous report, on a different pathology, where mice subjected to infection (strong inflammatory process) have an increase in their circulatory KC level [48]. The finding highlights that, when various cells (large cell type range from immune cells to tumor cells) contribute to the secretion of a cytokine-like KC, the sex difference is not as obvious as in prior mentioned cytokines.

Our own experience has shown, whether in human samples or in mouse melanoma models [49], that circulatory cytokines have different patterns matching the cutaneous melanoma stages and most probably the clinical evolution of the patients [15, 50, 51].

The sex differences that we present herein in terms of cytokine/chemokine circulatory pattern can highlight several aspects. Prior studies regarding circulatory immune cells in C57 BL/6 male versus females were published [52]. It was shown that in this strain, significant circulatory lymphocyte and monocyte population difference was reported. C57 BL/6 males have lower lymphocyte count in comparison to females, while higher monocyte values in males versus females, findings that are not age-dependent. Thus, at least for activated lymphocytes and monocytes that secrete IL-1, IL-6, IL-12, and TNF, the presented sex difference can be explained by these immune cell dissimilarities. We highlight that, as reported, sex hormones impact on gene expression in immune cells and lead to sexually dimorphic immune responses [37]. Another aspect that emerges from our study is that touching upon the intervention of neuroendocrine factors in the development of skin cancers [53–55], several intracellular pathways support the connection between estrogens, estrogen receptors, and melanoma. While estrogen receptor-beta plays an antiproliferative role, alpha type promotes cell growth and cellular atypia [55]. Thus, further studies of melanoma development in females comparative with males would open a new area of investigation for (immune) therapy efficiency evaluation.

The wide variety of circulatory mediators and cells involved in the complex switch between acute and chronic inflammation could provide new early indicators of the tumor immunosuppressive status correlated with melanoma progression.

## 5. Conclusion

We aimed in this study to monitor soluble cyto/chemokine production as possible markers for disease evolution in cutaneous melanoma animal model. We found important differences in healthy mice in terms of cytokine pattern associated to sex. Moreover, even the melanoma evolution in both sexes in terms of cytokine types was different; more so, if low doses of cytostatics were used as therapy agent these differences were enhanced. With this study, we aim to point out that the inflammatory status of experimental melanoma models is associated with the animal sex and should be considered as such. Another conclusion that can be drawn from the findings is that when evaluating in humans, the circulatory cytokine levels as markers for cutaneous melanoma, gender association should be taken into account and the link between the endocrine and immune system can shed new light in disease prognosis. Therefore, evaluation of (immune) therapy efficiency in patients, female versus male, should allow for immune status particularities guiding the overall disease management.

## Figures and Tables

**Table 1:** Cytokine/chemokine panel analyzed by Luminex-based cytokine bead array.

Function	Name	Cell source
Proinflammatory cytokines	IL-1-beta, IFN-gamma	Lymphocytes
	IL-12 (p70)	Monocytes/macrophages and B cells
	TNF-alpha	Macrophages

Anti-inflammatory cytokines	IL-10	Mainly monocytes

Pleiotropic cytokines	GM-CSF	Macrophages, T cells, mast cells, natural killer cells, endothelial cells, and fibroblasts
	IL-6	Mainly T cells and macrophages

Chemokines	MCP-1	Primarily monocytes, macrophages, and dendritic cells
	MIP-1	Memory CD8^+^ T cells
	KC	Macrophages, neutrophils, epithelial cells, and melanoma cells
